# Three new cavernicolous species of the millipede genus *Trichopeltis* Pocock, 1894 from southern China (Diplopoda, Polydesmida, Cryptodesmidae)

**DOI:** 10.3897/zookeys.710.20025

**Published:** 2017-10-19

**Authors:** Weixin Liu, Sergei Golovatch, Mingyi Tian

**Affiliations:** 1 Department of Entomology, College of Agriculture, South China Agricultural University, 483 Wushanlu, Guangzhou 510642, China; 2 Institute for Problems of Ecology and Evolution, Russian Academy of Sciences, Leninsky pr. 33, Moscow 119071, Russia

**Keywords:** *Trichopeltis*, new species, key, troglobite, southern China

## Abstract

Three new species of *Trichopeltis* are described from caves in southern China: *T. bellus* **sp. n.**, *T. intricatus* **sp. n.**, and *T. reflexus* **sp. n.**, all presumed troglobites. The former two come from Yunnan Province, the latter one from Hunan Province. An updated key to all eleven currently known species of *Trichopeltis* is provided.

## Introduction

The Cryptodesmidae is a relatively small millipede family that globally encompasses approximately 40 genera and 130 species. It is distributed from Mexico to Argentina in the Americas, occurring also in tropical Africa and tropical to subtropical Asia to Papua New Guinea and Japan in the East (Enghoff et al. 2015). In tropical or subtropical Asia and Australasia, Cryptodesmidae currently comprise only 12 genera (including two that remain dubious) and 36 species.

At present, the Indo-Malayan genus *Trichopeltis* Pocock, 1894 is composed of eight species: *T. bicolor* (Pocock, 1894), the type species from Sumatra, Indonesia; *T. cavernicola* Golovatch, 2016 and *T. muratovi* Golovatch & VandenSpiegel, 2017, both from Laos; *T. doriae* Pocock, 1895 and *T. feae* Pocock, 1895, both from Myanmar; *T. kometis* (Attems, 1938) (= *T. deharvengi* Golovatch, Geoffroy, Mauriès & VandenSpiegel, 2010) from Vietnam, Laos and Cambodia; *T. latellai* Golovatch, Geoffroy, Mauriès & VandenSpiegel, 2010 from Guizhou Province, China; and *T. watsoni* Pocock, 1895 from Myanmar and Darjeeling District, India. *Trichopeltis latellai* is also the only genus and species of Cryptodesmidae reported so far from China (Golovatch and VandenSpiegel 2017).

Rather recently, *Trichopeltis* has been reviewed and a key provided to five of its species (Golovatch et al. 2010, Golovatch and Akkari 2016, and the references therein). The present paper records an additional three new species of *Trichopeltis*, all three of which are presumed to be troglobites from southern China.

## Materials and methods

All specimens used in this study were collected by hand from caves in southern China and are preserved in 95% ethanol. The type material is deposited in the zoological collection of the South China Agricultural University, Guangzhou, China (SCAU).

Observations and dissections were performed using a Leica S8 APO stereo microscope. The line drawings were prepared with a Zeiss Imager Axioskop40 microscope and a camera lucida attached for the scope. Photographs were taken with a Canon EOS 40D camera, then focus-stacked with Z-stack software, or Keyence VHX-5000 digital microscope, and further edited using Adobe Photoshop CS5 and Illustrator CC software.

The terminology used here follows that of Golovatch et al. (2012) and Golovatch and VandenSpigel (2017).

## Taxonomy

### Trichopeltis bellus sp. n.

Taxon classificationAnimaliaMicrothyrialesTrichothyriaceae

http://zoobank.org/12B62C80-9542-458B-B5BC-7420F16729CA

Figs 1 , 2 , 3

#### Type material.

Holotype ♂ (SCAU), China, Yunnan Province, Qujing City, Luoping County, Machang Village, Shuiyuan Dong Cave, 24°49'33"N, 104°21'48"E, 1530 m, 18.VI.2015, leg. Mingyi Tian, Weixin Liu, Xinhui Wang & Mingruo Tang.

#### Paratypes.

2 ♀ juv. (SCAU), same data as the holotype.

#### Etymology.

To emphasize the very pretty appearance of this species; adjective.

#### Diagnosis.

**Figure 1.**
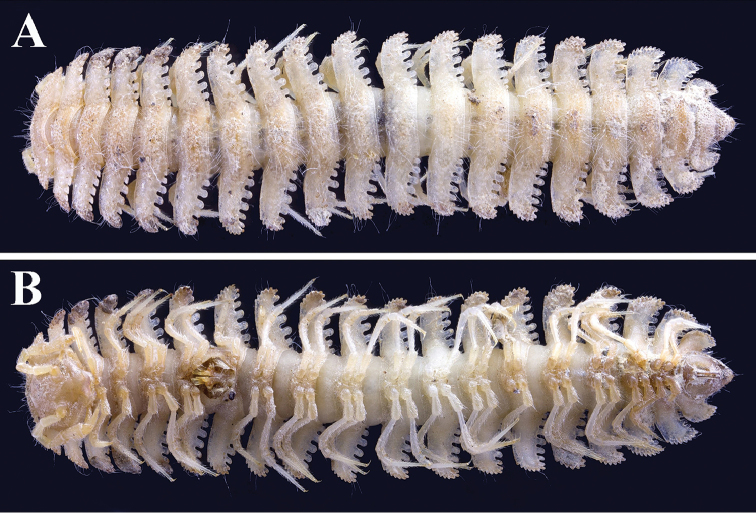
*Trichopeltis bellus* sp. n., ♂ holotype. **A–B** habitus, dorsal and ventral views, respectively.

Differs from other species of the genus by the unusually elongate and densely setose gonopodal coxa. Superficially similar to *T. intricatus* sp. n., but distinguished from the latter in the longer tergal setae (Fig. 1A), and gonopodal femorite with a large, club-shaped, mesoventral lobe (Fig. 3). See also Key below.

#### Description.

**Figure 2.**
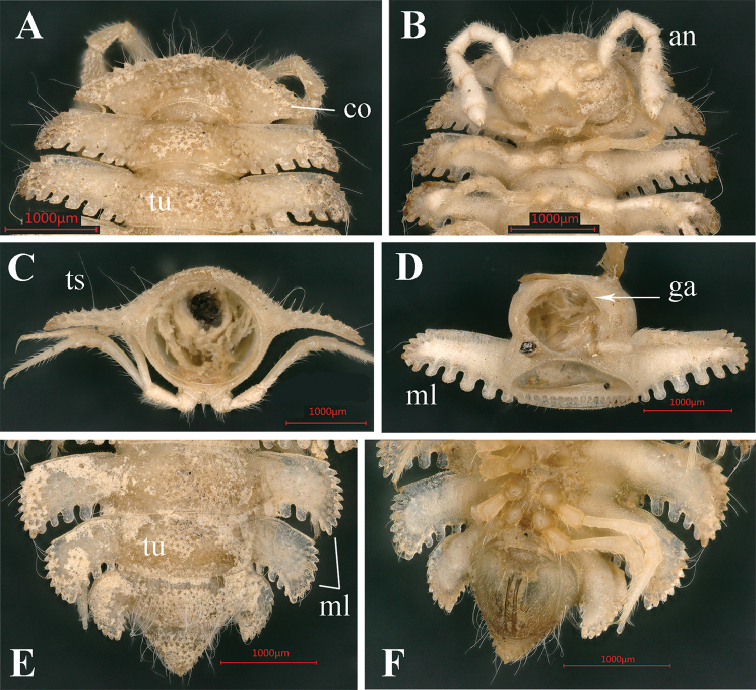
*Trichopeltis bellus* sp. n., ♂ holotype. **A** collum and segments 2–3, dorsal view **B** head and segments 1–4, ventral view **C** cross-section of segment 8, caudal view **D** segment 7, ventral views **E–F** segments 17–19 and telson, dorsal and ventral views, respectively. Abbreviations: an = antenna; co = collum; ga = gonopod aperture; ml = marginal lobules; ts = tergal seta; tu = tubercles.

Length of holotype *ca.* 16 mm, width of midbody pro- and metazonae 1.5 and 4.5 mm, respectively. Coloration in alcohol uniformly light yellow. Adults with 20 segments (Fig. 1). In width, head < collum < segment 2 < 3 < 4 < 5 < 7–16 < 6 (Figs 1A, 2A); following segment 16, body rapidly tapering towards telson (Fig. 1A).

Head: vertex densely pilose and microgranulate, clypeus clearly smooth (Fig. 2B), epicranial suture superficial. Labrum with three teeth. Antennae short and clavate, reaching behind segment 2 when stretched dorsally; in length, antennomere 6 > 3 > 2 > 5 = 4 > 1 > 7 (Fig. 2B).

Collum fan-shaped (Fig. 2A), incompletely covering the head from above, dorsal surface with six irregular transverse rows of small, round, setigerous tubercles (Fig. 2A). Marginal lobules on collum: 13+13 small, microvillose, setigerous, nearly sharp anteriorly and 6+6 similarly small, microvillose, but squarish laterally.

Mid-dorsal regions on segments 2–16 with five more or less regular, transverse rows of similarly small, setigerous tubercles, 6–8 + 6–8 per row (Fig. 2A). The tubercles extending onto paraterga, but each of the latter only with three or four irregular rows of similar tubercles (Fig. 1A). Following metaterga with 6–8 rows of smaller tubercles (Fig. 2E).

**Figure 3.**
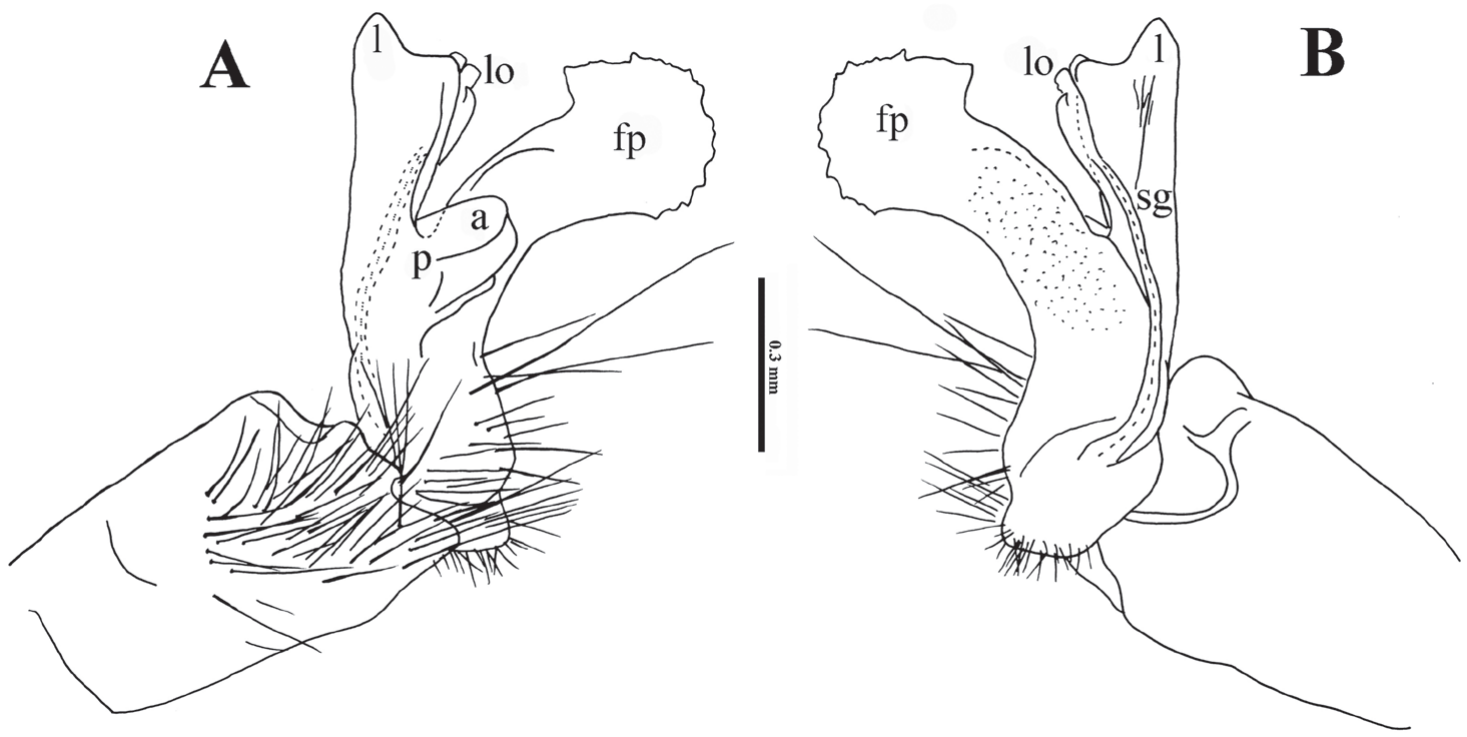
*Trichopeltis bellus* sp. n., ♂ holotype. **A–B** right gonopod, lateral and mesal views, respectively. Abbreviations: a = lobe on acropodital process; fp = femoral process; l = apical lobe; lo = lobules; p = acropodital process; sg = seminal groove.

Paraterga very strongly developed (Figs 1–2), high, only slightly declivous, but never extending down below level of venter (Fig. 2C), each with 6–8 small, dentiform, lateral and 5–7 much larger, squarish caudolateral lobules, all evident, setigerous and microvillose (Figs 1–2). Caudolateral lobules on paraterga mostly oblong, relatively large, and well separated from one another (Figs 1–2). Caudolateral corner of paraterga projecting behind rear tergal margin only on segments 17–19 (Fig. 3E–F).

Integument clearly microgranulate throughout (Fig. 1A), prozonae finely alveolated. Limbus regularly crenulated. Stricture between pro- and metazonae broad, shallow and finely microgranulated. Tergal setae simple, very long and subfiliform (Fig. 1A). Ozopores invisible, pore formula untraceable.

Epiproct tip sharp, with four spinnerets apically (Fig. 2F). Hypoproct subtrapeziform, 1+1 caudal setigerous papillae clearly separated (Fig. 2F).

Pleurosternal carinae clearly present on segment 2 alone. Sterna modestly setose, cross-impressions moderate, clearly broadened between ♂ coxae 6, 7 and 9 (Figs 1B, 2D). Gonopod aperture rhomboid (Figs 1B, 2D).

Legs very long and slender, unmodified, produced beyond paratergal lateral margin (Figs 1B, 2C), about 1.8 times as long as midbody height.

Gonopods (Fig. 3) complex. Coxa subcylindrical, unusually long, and very densely setose on lateral side. Prefemora densely setose, with a few particularly long setae. Femorite composed of extremely strong mesoventral process (**fp**), the latter about as long as telopodite, slightly curved, club-shaped. Acropodite suberect, laterally with a smaller, parabasal, rounded process (**p**) supporting a still smaller lobe (**a**) apically. Acropodite with one evident apical lobe (**l**) and a few very small subapical lobules (**lo**). Seminal groove (**sg**) entirely mesal, terminating without pulvillus at **lo**, forming no distinct solenomere.

#### Remark.

Based on the unpigmented body and long legs, this species is probably a troglobite.

### Trichopeltis intricatus sp. n.

Taxon classificationAnimaliaMicrothyrialesTrichothyriaceae

http://zoobank.org/2CFBA46B-60D9-4798-B8A2-5C373BA620EF

Figs 4 , 5 , 6

#### Type material.

Holotype ♂ (SCAU), China, Yunnan Province, Kunming City, Shilin County, Guishan Town, Haiyi I Dong Cave, 24°38'50"N, 103°32'49"E, 1890 m, 16.VI.2015, leg. Mingyi Tian, Weixin Liu, Xinhui Wang & Mingruo Tang.

#### Etymology.

To emphasize the complex gonopods; adjective.

#### Diagnosis.

**Figure 4.**
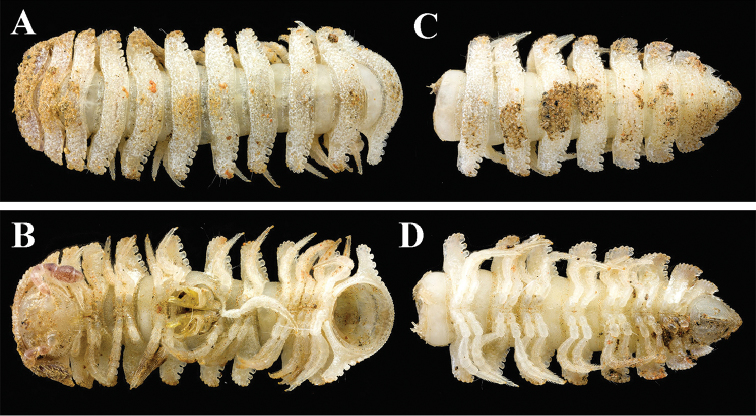
*Trichopeltis intricatus* sp. n., ♂ holotype. **A–B** anterior part of body **C–D** posterior part of body, dorsal and ventral views, respectively.

Differs from all congeners except *T. bellus* sp. n. by the unusually densely setose gonopodal coxa, and from all species by the particularly complex gonopod which shows a number of peculiar processes and lobules (Fig. 4). See also the Key below.

#### Description.

Length of holotype *ca.* 10 mm, width of midbody pro- and metazonae 1.5 and 2.5 mm, respectively. Coloration in alcohol nearly pallid. Body with 20 segments (Fig. 4). All characters as in the previous species (Figs 1–3), except as follows. In width, head < collum < segment 2 < 3–4 < 5 < 6 < 7; thereafter body increasingly tapered towards telson (Fig. 4).

**Figure 5.**
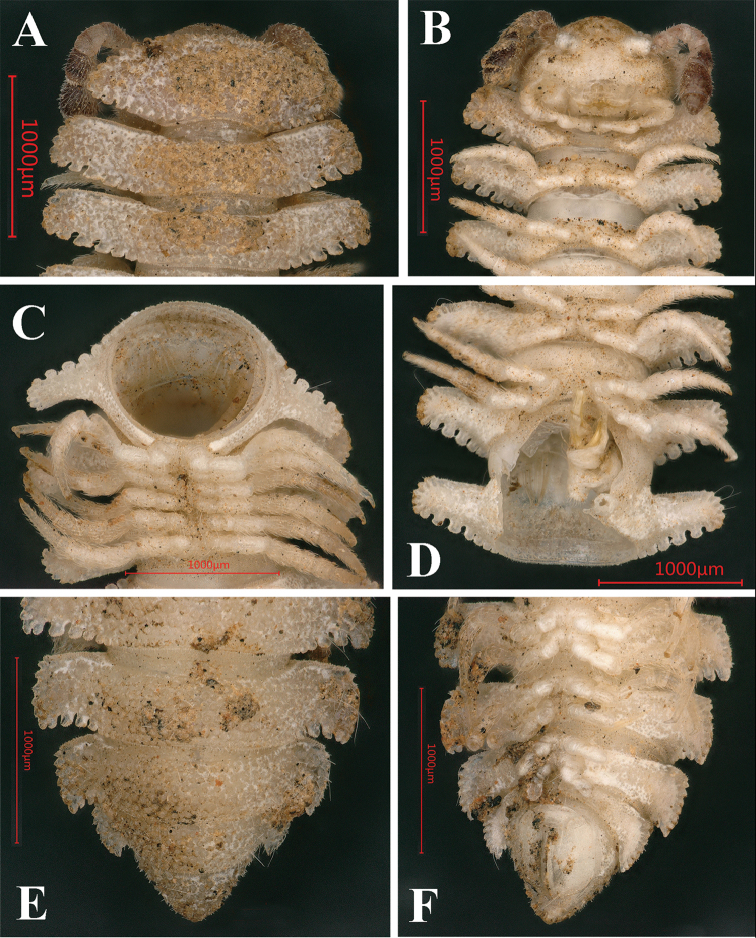
*Trichopeltis intricatus* sp. n., ♂ holotype. **A** collum and segments 2–3, dorsal view **B** head and segments 1–4, ventral view **C** cross-section of segment 10, caudal view **D** segment 5–7, ventral views **E–F** segments 16–19 and telson, dorsal and ventral views, respectively.

Head sparsely pilose. Antennae very short and clavate, reaching behind segment 2 when stretched dorsally; in length, antennomere 6 > 3 > 4 = 5 = 2 = 7 = 1 (Fig. 5B).

Collum fan-shaped, inverted subtrapeziform, incompletely covering the head from above, with five irregular transverse rows of small, round, setigerous tubercles (Fig. 5A). Marginal lobules on collum: 15+15 small, microvillose, nearly sharp anteriorly and 6+6 similarly small, but squarish laterally.

Mid-dorsal regions on segments 2–16 with five regular, transverse rows of about 15+15 similarly small, setigerous tubercles extending onto paraterga, in frontal and caudal rows smaller than others (Fig. 4A & C).

Paraterga 3–5 with 4–5 small, dentiform, lateral and 5–6 much larger, squarish, caudal lobules. Similarly, paraterga 2 and 6–16 with 6 lateral, 6–7 caudal lobules.

Tergal setae simple, very short and mostly abraded (Fig. 4A & C).

Epiproct short, conical (Fig. 5D).

Gonopod aperture subcordiform (Figs 4B, 5D).

Legs short and robust (Figs 4–5), produced beyond paratergal lateral margin, about 1.2 times as long as midbody height.

**Figure 6.**
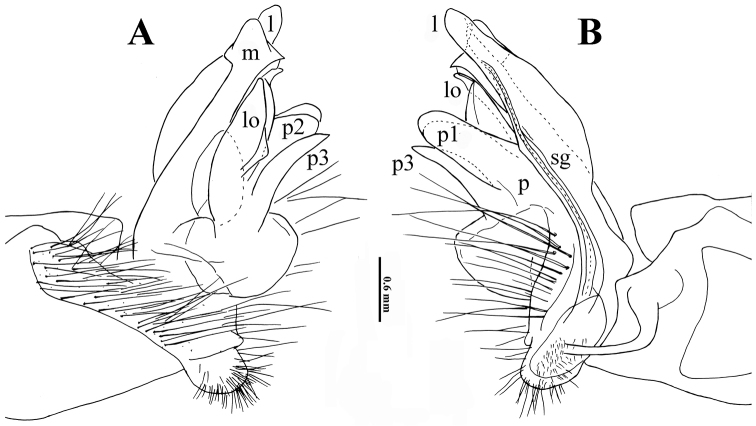
*Trichopeltis intricatus* sp. n., ♂ holotype. **A–B** right gonopod, lateral and mesal views, respectively. Abbreviations: l = apical lobe; lo = lobules; m = mushroom-shaped lobe; p = acropodital process; p1–3 = processes 1–3; sg = seminal groove.

Gonopods (Fig. 6) very complex. Coxa short and squarish, but unusually densely setose laterally, much like in the previous species. Prefemora densely setose, but with more numerous longer setae. Femorite only slightly curved caudally at base with a clearly tripartite femoral process (**p**), branches **p1** (mesal) and **p2** (lateral) being subequal, long and rounded at end, branch **p3** being basalmost slender and acuminate at end. Acropodite longer than **p**, at base with a long, slender, apically mushroom-shaped lobe (**m**) on lateral side, and an even longer, slender, finger-shaped, mesal, apical lobe (**l**), as well as a group of lobules (**lo**) between **p** and **m**. Seminal groove (**sg**) entirely mesal, terminating without pulvillus near **lo**, forming no distinct solenomere.

#### Remark.

Based on the pallid body, this species may be a troglobite.

### Trichopeltis reflexus sp. n.

Taxon classificationAnimaliaMicrothyrialesTrichothyriaceae

http://zoobank.org/63B2C168-31B7-4631-AD90-304AD18105B0

Figs 7 , 8 , 9

#### Type material.

Holotype ♂ (SCAU), China, Hunan Province, Chenzhou City, Linwu County, Xianghualing Town, II Dong Cave, 19.VI.2009, leg. Mingyi Tian & Zhihong Xue (CHIhn09-LWX03).

#### Paratypes.

1 ♂, 3 ♀ (SCAU), same data as the holotype.

#### Etymology.

To emphasize that most of the paraterga are upturned.

#### Diagnosis.

Differs from all congeners except *T. cavernicola* Golovatch, 2016 by the clearly upturned paraterga, and from all congeners by the shapes of the various lobes which are all confined to the distal third of the gonopodal telopodite. Among congeners, only *T. latellai* Golovatch, Geoffroy, Mauriès & VandenSpiegel, 2010, from two caves in Guizhou Province (Golovatch et al. 2010) strongly resembles *T. reflexus* sp. n. in showing a similarly condensed apical third of the gonopodal telopodite, but that in the latter species is less strongly curved, untwisted and more elaborate. See also Key below.

#### Description.

**Figure 7.**
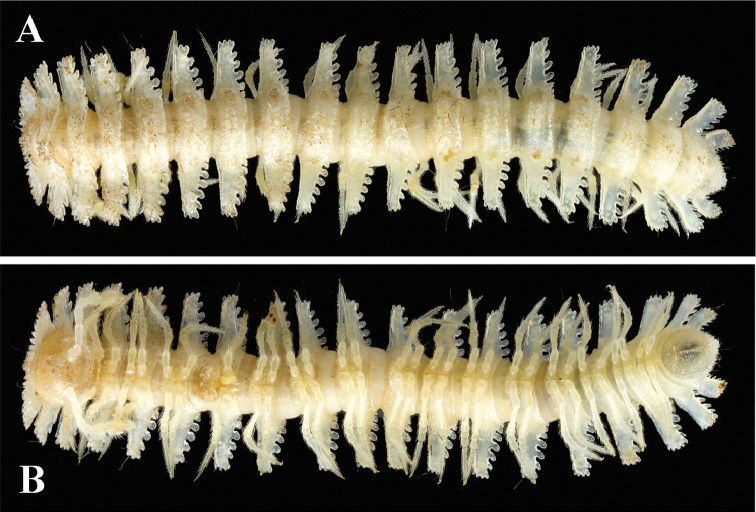
*Trichopeltis reflexus* sp. n., ♂ paratype. **A–B** habitus, dorsal and ventral views, respectively.

Length of both sexes *ca.* 10 mm, width of midbody pro- and metazonae 0.8 and 2.5 (♂) or 1.0 and 2.5 mm (♀), respectively. Coloration in alcohol nearly pallid. Body with 20 segments (Fig. 7). All characters as in *T. bellus* sp. n. (Figs 1–3), except as follows. In width, head < collum < segment 2 < 4 < 3 = 5 < 6–15; thereafter body increasingly tapered towards telson (Fig. 7).

**Figure 8.**
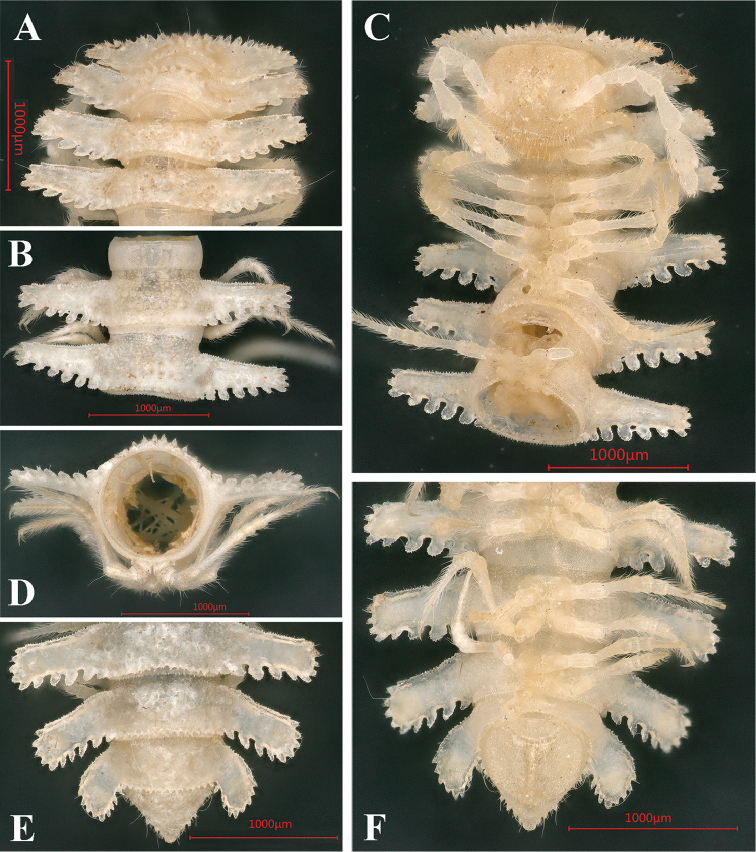
*Trichopeltis reflexus* sp. n., ♂ paratype. **A** collum, segments 2–4, dorsal view **B** segments 8–9, dorsal view **C** head and segments 1–7, ventral view **D** cross-section of segment 9, caudal view **E–F** segments 17 or 16–19 and telson, dorsal and ventral views, respectively.

Collum with 3–4 irregular transverse rows of small and sharpened tubercles. Marginal lobules on collum: 13+13 small, setigerous, nearly sharp anteriorly and 3+3 similarly small, dentiform laterally (Fig. 8A–B).

Mid-dorsal regions on segments 2–16 with two regular, transverse rows of 3+3 and 4+4 tubercles similar to those on collum (Fig. 8A–B), extending onto paraterga, the latter with 2–3 similar tubercles; following metaterga with three rows of 3+3, 2+2 and 3+3 tubercles (Figs 7A, 8E–F). Caudal margin of mid-dorsal region of metaterga with 12–16 lobules (Fig. 8A–B, E).

Paraterga very strongly developed (Figs 7–8), lateral margin narrow and upturned, but still remaining below a regularly convex dorsum (Fig. 8D). Paraterga with 3–4 lateral and 4–6 caudal lobules (Figs 7–8).

Tergal setae simple and short, mostly abraded (Fig. 7A).

Epiproct short, conical (Fig. 8F).

Pleurosternal carinae poorly-developed, but present on segments 2 and 3.

Sterna clearly broadened only between ♂ coxae 9. Gonopod aperture suboval (Fig. 8C).

Legs short, but slender, about 1.2 times as long as midbody height (Figs 7–8).

**Figure 9.**
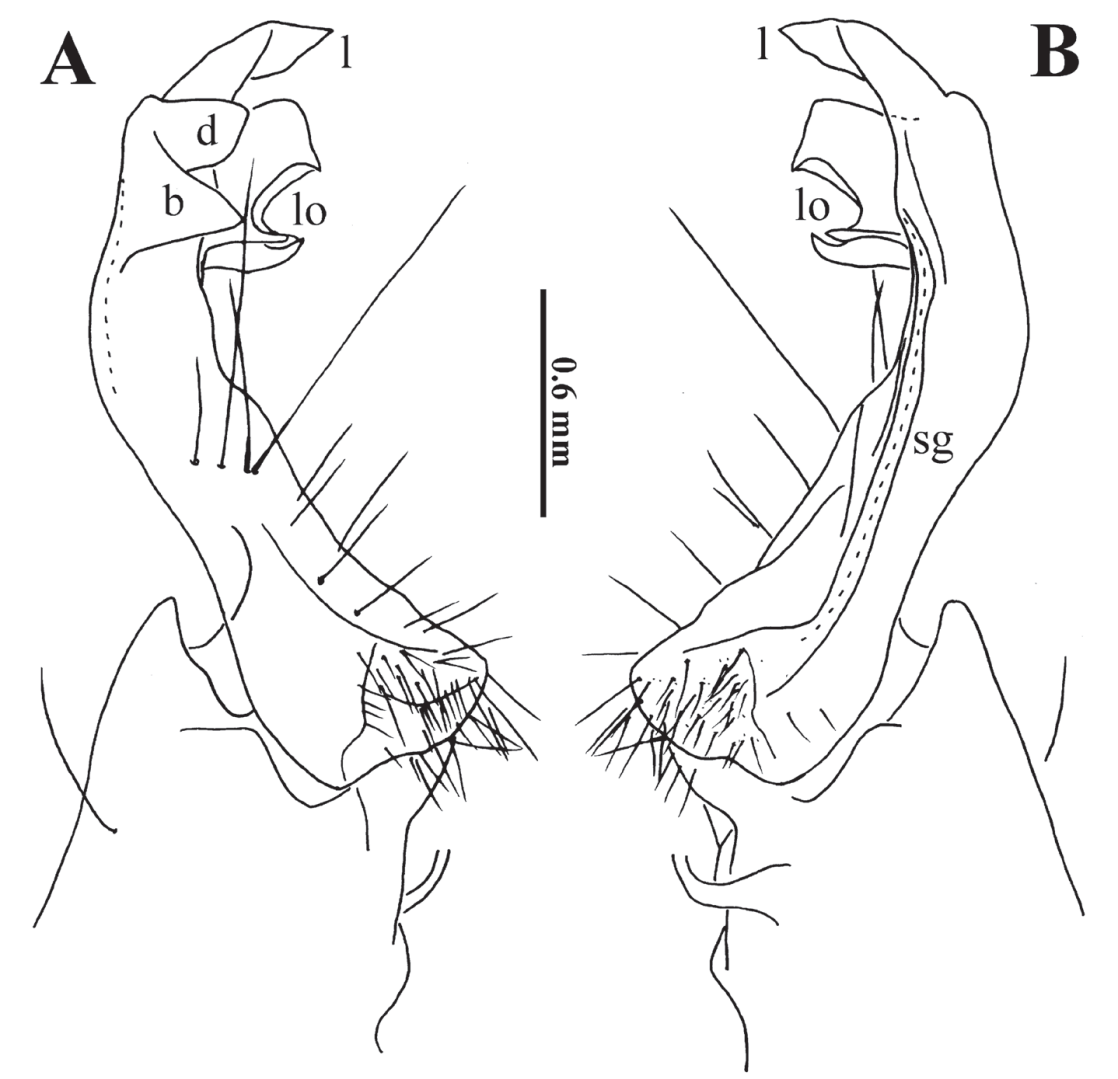
*Trichopeltis reflexus* sp. n., ♂ paratype. **A–B** right gonopod, lateral and mesal views, respectively. Abbreviations: b = acropodite basal lobe; d = acropodite distal lobe; l = apical lobe; lo = lobules; sg = seminal groove.

Gonopods (Fig. 9) complex only in apical third of telopodite. Coxa as usual, short and squarish, with one long seta. Prefemoral part as usual, with only a few particularly long setae distally. Telopodite slightly curved caudally, without femoral processes at base. Acropodite strongly condensed, tripartite, with a large, subtriangular, more basal lobe (**b**) and a short, squarish, more distal lobe (**d**), both similar in size and lying on lateral side; caudal to both **b** and **d** with a few differently shaped lobules (**lo**); apical lobe (**l**) highest, acuminate, folded. Seminal groove (**sg**) entirely mesal, terminating without pulvillus near **lo**, forming no distinct solenomere.

#### Remark.

Based on the pallid body and slender legs, this seems to be a troglobite.

### Key to species of *Trichopeltis*

(modified after Golovatch et al. 2010 to incorporate all five species described since the latest synopsis)

**Table:** 

1	Tegument unpigmented, pallid to light yellowish; cavernicolous species	**2**
–	Tegument clearly pigmented, red- or grey-brown to blackish; epigean species	**6**
2	Central parts of metaterga with 2–4 irregular transverse rows of setigerous tubercles; gonopodal coxa as usual, at most with only few setae	**3**
–	Central parts of metaterga with 5–6 irregular transverse rows of setigerous tubercles; gonopodal coxa unusually densely setose on lateral side (Figs 3, 6); Yunnan, China	**5**
3	Paraterga declivous; tergal setae very long, about half as long as body diameter; gonopodal telopodite clearly twisted; Guizhou, China	***T. latellai***
–	Paraterga clearly upturned; tergal setae much shorter; gonopodal telopodite untwisted, seminal groove running entirely on mesal side	**4**
4	Central parts of metaterga with 3–4 irregular transverse rows of setigerous tubercles; gonopodal telopodite with a pulvillus subapically; Laos	***T. cavernicola***
–	Central parts of metaterga with 2–3 rather regular transverse rows of setigerous tubercles (Fig. 7A); gonopodal telopodite without pulvillus (Fig. 9); Hunan, China	***T. reflexus* sp. n.**
5	Tergal setae very long (Figs 1–2); gonopods relatively simple (Fig. 3)	***T. bellus* sp. n.**
–	Tergal setae very short (Figs 4–5); gonopods especially elaborate	***T. intricatus* sp. n.**
6	Central parts of metaterga with 4–6 irregular transverse rows of setigerous tubercles	**7**
–	Central parts of metaterga with 2–3 irregular transverse rows of setigerous tubercles	**10**
7	Gonopodal telopodite clearly 3-branched, solenomere long and slender; Myanmar	***T. doriae***
–	Gonopodal telopodite without long branches, only more or less deeply notched apically; solenomere rudimentary, barely visible	**8**
8	Central parts of metaterga with 4–5 rather regular transverse rows of setigerous tubercles; gonopodal telopodite with a conspicuous accessory seminal chamber and a pulvillus, but devoid of denticles laterally or mesally; Laos	***T. muratovi***
–	Central parts of metaterga with 5–6 rather regular transverse rows of setigerous tubercles; gonopodal telopodite without accessory seminal chamber, but with a pulvillus, also abundantly denticulate either laterally or mesally	**9**
9	Body *ca.* 12 mm long and 3.0 mm wide; gonopodal telopodite abundantly denticulate on lateral face. Vietnam, Laos and Cambodia and possibly endemic to the Indochina Peninsula	***T. kometis***
–	Body *ca.* 16 mm long and 4.8 mm wide; gonopodal telopodite abundantly denticulate on mesal face. Sumatra, Indonesia	***T. bicolor***
10	Frontal margin of paraterga abundantly lobulated. Solenomere lobe-shaped, tip nearly pointed	***T. feae***
–	Frontal margin of paraterga entire, not lobulated. Solenomere axe-shaped, tip pointed	***T. watsoni***

## Conclusions and discussion

The family Cryptodesmidae was hitherto known to encompass three presumed troglobiont species: *Peridontodesmella alba* Schubart, 1957, from Brazil (Trajano et al. 2000); *Trichopeltis latellai*, from two caves in Guizhou, China (Golovatch et al. 2010); and *T. cavernicola*, from two caves in Laos (Golovatch 2016, Golovatch and VandenSpiegel 2017). The three new species described above show clear traits of troglomorphism, thereby considerably increasing the number of presumably troglobiont cryptodesmids known globally.

Almost all of southern China is blanketed by Earth’s most extensive karsts (Deharveng and Bedos 2012). Some of them are known to be especially rich in biodiversity, while the Mulun and surrounding karsts in Guangxi Province host some of the richest cave fauna of China (Deharveng et al. 2008). This fauna encompasses millipedes as well (Golovatch 2015).

At present, most of the species of *Trichopeltis*, including both epigean and cavernicolous taxa, occur in Indo-Burma and Indochina. With the description of the above three new taxa, and with further explorations of southern China karst region, the southern part of the country will certainly become an important hotspot of *Trichopeltis* diversity. Due to a rapid discovery of new species, the previous key (Golovatch et al. 2010), which is only a few years old, is already out of date. The same is likely to occur with the new key provided above, as there is little doubt that new taxa will be found in the near future at least in southern China.

## Supplementary Material

XML Treatment for Trichopeltis bellus

XML Treatment for Trichopeltis intricatus

XML Treatment for Trichopeltis reflexus
